# Is Moving More Memorable than Proving? Effects of Embodiment and Imagined Enactment on Verb Memory

**DOI:** 10.3389/fpsyg.2016.01010

**Published:** 2016-06-30

**Authors:** David M. Sidhu, Penny M. Pexman

**Affiliations:** Department of Psychology, University of CalgaryCalgary, AB, Canada

**Keywords:** simulation, embodiment, verb meaning, memory, semantic richness

## Abstract

Theories of embodied cognition propose that sensorimotor information is simulated during language processing (e.g., Barsalou, 1999). Previous studies have demonstrated that differences in simulation can have implications for word processing; for instance, lexical processing is facilitated for verbs that have relatively more embodied meanings (e.g., Sidhu et al., 2014). Here we examined the effects of these differences on memory for verbs. We observed higher rates of recognition (Experiments 1a-2a) and recall accuracy (Experiments 2b-3b) for verbs with a greater amount of associated bodily information (i.e., an embodiment effect). We also examined how this interacted with the imagined enactment effect: a memory benefit for actions that one imagines performing (e.g., Ditman et al., 2010). We found that these two effects did not interact (Experiment 3b), suggesting that the memory benefits of automatic simulation (i.e., the embodiment effect) and deliberate simulation (i.e., the imagined enactment effect) are distinct. These results provide evidence for the role of simulation in language processing, and its effects on memory.

## Introduction

Embodied cognition is a broad proposal for cognitive science that has taken several different forms (e.g., Barsalou, 2008; Wilson and Golonka, 2013; Glenberg, 2015). A core feature of the embodied cognition proposal is that sensorimotor experience is involved in a variety of cognitive processes. Importantly, sensorimotor experience can play a role in cognition even in the absence of external stimuli or movements, in the form of internal *simulations* of these experiences (Barsalou, 1999, 2008). According to Barsalou's (1999) *Perceptual Symbol Systems* theory, sensory experiences of a particular concept become organized and stored as a *simulator*. For instance, one's experiences viewing a car from multiple angles, hearing a car's engine, feeling a car's vibration, pressing the gas pedal, are all stored as part of the concept *car*, as *perceptual symbols*. Then, one can *simulate* a car, drawing on some combination of its constituent perceptual symbols to recreate the perceptual experience of a car. Studies have demonstrated that perceiving an object automatically elicits a simulation of interacting with it (e.g., Tucker and Ellis, 1998), and that seeing an action elicits a simulation of performing that action (e.g., Buccino et al., 2001). These simulations are similar to the actions themselves and involve similar brain areas (Gallese, 2007).

These sensorimotor simulations can also be evoked during the processing of language stimuli. This is consistent with the proposal that information gained through sensorimotor or bodily experience is important to the representation of word meaning, and that retrieving a word's meaning involves a simulation of that experience (e.g., Barsalou, 1999). Stanfield and Zwaan (2001) demonstrated that participants were faster to verify that an object had been mentioned in a previous sentence if its visual appearance matched what was implied by the sentence (e.g., faster to respond to an *open* umbrella if the sentence mentioned it being used during a storm). This suggests that readers create vivid simulations of a text's visual features. Similarly, Glenberg and Kaschak (2002) found that participants were faster to perform either a push or a pull movement, if it was congruent with the action described in a sentence (i.e., the action compatibility effect; cf. Papesh, 2015). This suggests that individuals also simulate the *actions* implied by a text. A neuroimaging study by Hauk et al. (2004) supported this notion, and demonstrated that this applies even when words are presented in isolation. They found that reading action words performed with the face, arm, or leg activated areas of the motor cortex responsible for moving those parts of the body.

A potential implication of these findings is that variations in the amount of simulation elicited by a word may lead to differences in processing (for a review, see Connell and Lynott, 2015). Indeed, studies have found differences in reaction time and accuracy when responding to words referring to objects that are more or less easy to interact with (i.e., the BOI effect; Siakaluk et al., 2008). This is consistent with sensorimotor simulation playing a role in word processing. Processing benefits for words eliciting a greater amount of simulation are consistent with theories of semantic richness and the notion that “more is better” (Balota et al., 1991, p. 214). Semantic richness is a multidimensional construct that quantifies the amount of meaning information associated with a given word (for a review, see Pexman, 2012). In general, words that are more semantically rich enjoy processing benefits on a variety of lexical tasks (e.g., Pexman et al., 2008; Yap et al., 2011). This is believed to be due to stronger semantic activation for semantically rich items.

While much of the research on semantic richness effects has examined noun stimuli, Sidhu et al. (2014) studied the implications of embodied semantic richness for verb processing. Verbs vary in the extent to which their meaning involves the human body: for instance, the human body is crucial to the meaning of the word *move*, but it has little to do with the meaning of the word *prove*. Sidhu et al. (2014) quantified these differences in relative embodiment by asking participants to rate the extent to which the meaning of each of nearly 700 verbs involved the human body. The authors found processing advantages for high embodiment verbs on a lexical decision task, a picture-naming task, and a syntactic categorization task. These findings were consistent with the notion that high embodiment verbs elicit a greater amount of simulation, and thus are processed faster as a result of this type of semantic richness. The purpose of the present study was to further examine potential differences in simulation between high and low embodiment verbs, by examining their implications for memory.

A basic principle in the memory literature is that differences in elaboration during encoding have consequences for later memory (Lockhart and Craik, 1990). That is, items encoded with richer or more extensive processing will be remembered better at a later time. Importantly, some studies have shown that features of the items themselves can lead to more or less elaborate encoding. For example, Hargreaves et al. (2012) found evidence that words with a greater number of semantic features were remembered better on a free recall task. This was interpreted as evidence that variations in the amount of information associated with an item can lead to variations in elaboration during encoding.

Several previous studies have examined differences in memory based on variations in the amount of *motor* information associated with a word. Madan and Singhal (2012) examined recall memory for words referring to objects that were highly manipulable (e.g., *camera*) as compared to those that were not (e.g., *table*). The authors also varied encoding instructions by asking participants to make a decision based on: their personal experience with the object, the manipulability of the object, or the number of letters in the word. Overall, participants were better able to remember the names of highly manipulable objects, though this pattern was reversed in participants who were explicitly asked about the manipulability of the objects. The authors interpreted the overall benefit for highly manipulable items as being due to automatic activation of motor representations. That is, the differences in motor processes activated by highly manipulable and non-manipulable objects may have led to a memory benefit for the former. This is consistent with the notion that differences in simulation can have consequences for memory. Notably, in the Madan and Singhal study, when participants deliberately processed items' motor features, this “automatic memory enhancing effect” of motor simulation (p. 1568) was superseded, suggesting that automatic simulation can be modulated by more deliberate processes.

Relatedly, a study by Montefinese et al. (2013) examined the relationship between verb embodiment and memory. They examined recognition memory for manipulatory verbs (i.e., verbs referring to actions performed with the hands; e.g., *sew*) as compared to non-manipulatory verbs (e.g., *sleep*). Results indicated that participants were more likely to categorize manipulatory verbs as previously seen, both for old and new items. Thus, both hit rates and false alarm rates were higher for manipulatory verbs. The authors' interpretation was that the similarity in evoked motor information between old and new manipulatory verbs may have led to more false remembering for new manipulatory verbs. Thus, these results also suggest that the simulation elicited by items during encoding can have effects on memory; however, those effects may not always be facilitory.

Other evidence for the role of simulation in memory has come from studies that have manipulated encoding conditions to vary the amount of motor information that participants evoke during encoding. The *enactment effect* is the finding is that individuals often have a better memory for action-noun phrases (e.g., *roll the ball*) that they themselves act out during encoding, as opposed to those that they watch someone else perform, or simply read about (Engelkamp and Krumnacker, 1980; Cohen, 1981). One explanation for this finding is that the additional motor information evoked as a result of pantomiming an action facilitates encoding and retrieval (Engelkamp, 1998; Nilsson, 2000). Another potential explanation is that preparing to perform an action necessitates more extensive processing of the action (Helstrup, 1986).

Interestingly, enactment effects have also been observed for imagined actions: differences in memory emerge when imagining oneself performing an action as compared to imagining someone else performing an action (Engelkamp et al., 1989; Denis et al., 1991). We will refer to these instances of the enactment effect, in which self-performed actions are imagined as opposed to overtly performed, as the “imagined enactment effect.” A version of this was recently investigated by Ditman et al. (2010), who presented participants with scenarios describing actions phrased in the first person (e.g., *I am rolling the ball*), the second person (e.g., *you are rolling the ball*) or the third person (e.g., *he/she is rolling the ball*). Previous research has demonstrated that readers will simulate a scene from different points of view depending on the pronoun used (Brunyé et al., 2009). As such, the authors expected better memory for the actions described in the second person scenarios (because reading: *you are rolling the ball*, should elicit a simulation of the self performing the action, from the reader's point of view) as compared to those in the first or third person. This was because second person scenarios should evoke a greater amount of motor information. This was borne out in their results, with higher *d*' scores (discriminability in recognition memory) for actions in the second person scenarios.

In the current study, we first examined the effect of variations in relative embodiment on memory (Experiments 1a and 1b). Our hypothesis was that the greater amount of simulation elicited by high embodiment verbs would lead to more elaborate encoding for those items, and thus better memory. Notably, embodiment was defined here in terms of the entire body (as in Sidhu et al., 2014), as opposed to previous studies that have examined verbs referring to actions performed with particular body parts (e.g., the hands in Montefinese et al., 2013). Second, to further explore the nature of this effect, we examined the joint effects of embodiment and imagined enactment, investigating whether verb embodiment would interact with the imagined enactment effect when participants were instructed to create mental simulations of the studied actions (Experiments 2a–3b). The results of previous studies (e.g., Madan and Singhal, 2012) have been taken to suggest that the memory benefit for items with more associated motor information is due to automatic simulation. If the embodiment effect is due to automatic simulation, then it may be distinct from the more deliberate, episodic simulation evoked in the imagined enactment effect, and imagined enactment and embodiment should thus have independent effects in our memory tasks. On the other hand, if embodiment effects and imagined enactment effects have the same source (some kind of generic simulation that is tapped by both embodiment and enactment) then we expected that they would interact in our memory tasks.

## Experiment 1

We examined recognition memory for verbs that were high and low in rated embodiment, in experiments run online (Experiment 1a) and in the laboratory (Experiment 1b).

### Experiment 1a

#### Method

##### Participants

This and subsequent studies were carried out in accordance with the University of Calgary Ethics Committee, with written informed consent from all subjects. All subjects gave written informed consent in accordance with the Declaration of Helsinki. Participants were 56 undergraduate students (52 female; *M* age = 20.52, *SD* = 3.83) at the University of Calgary who participated for bonus credit in a Psychology course. All participants in this and the following experiments had normal or corrected-to-normal vision, and reported English proficiency. The University of Calgary Conjoint Faculties Research Ethics Board approved the experiments described here.

##### Stimuli and Procedure

Stimuli were 140 verbs selected from the embodiment norms collected by Sidhu et al. (2014). These verbs were separated into two lists of 70 items, each containing 35 verbs rated high in embodiment (i.e., a rating > 3.5 on a seven point scale) and 35 verbs rated low in embodiment (i.e., a rating < 3.5). Within each list, verbs high and low in embodiment were matched in terms of length, log transformed HAL word frequency, number of morphemes (Balota et al., 2007), orthographic Levenshtein distance (Yarkoni et al., 2008), imageability (Chiarello et al., 1999), and age of acquisition (Kuperman et al., 2012); they did, however, differ in terms of their rated embodiment (Sidhu et al., 2014), see Table 1. Across lists, high and low embodiment verbs were also matched on all of these variables. See Appendix A for a full list of verb stimuli. The stimuli also included 70 nouns that were matched to both lists of verbs on these variables (except for relative embodiment).

**Table 1:** **Mean values (standard deviations in parentheses) of lexical and semantic variables for high and low embodiment verbs, Experiments 1a and 1b**.

	**High embodiment verbs**	**Low embodiment verbs**	***t***	***p***	**Cohen's *d***
Length	5.71 (1.39)	5.77 (1.23)	0.26	0.80	0.05
Log HAL frequency	8.08 (1.78)	8.31 (1.58)	0.79	0.43	0.14
Number of morphemes	1.44 (0.56)	1.43 (0.55)	0.15	0.88	0.02
OLD	1.97 (0.41)	2.00 (0.41)	0.46	0.65	0.07
Imageability	409.13 (61.56)	393.43 (64.40)	1.47	0.14	0.25
AoA	8.43 (2.27)	8.86 (2.16)	1.16	0.25	0.19
Embodiment	4.37 (0.48)	2.79 (0.26)	24.25	<0.001	4.27

*OLD, Orthographic Levenshtein distance; AoA, Age of Acquisition*.

Participants took part in Experiment 1a online, through the research tool Qualtrics. During the study phase, participants saw one list of 70 verbs, and the list of 70 nouns, one item at a time, with items intermixed in a random order. Their task was to make a response via mouse-click every time they saw a verb, but to withhold a response if they saw a noun. We used this encoding task to ensure that participants processed the meaning of the verbs. Regardless of whether or not participants made a response, a blank screen replaced each item after 5000 ms. This blank screen was shown for 1000 ms, after which the next trial began. Verbs were presented in their base form (e.g., *run*).

Following the study phase, participants were asked to solve as many addition problems as they could in 5 min, after which they took part in an unexpected recognition test. During this test, participants saw both lists of 70 verbs (i.e., the list of verbs that they had previously seen, and the list of verbs that they hadn't seen), one item at a time, intermixed in a random order. They indicated whether they thought each verb was old or new via mouse-click.

#### Results

**Figure 1:**
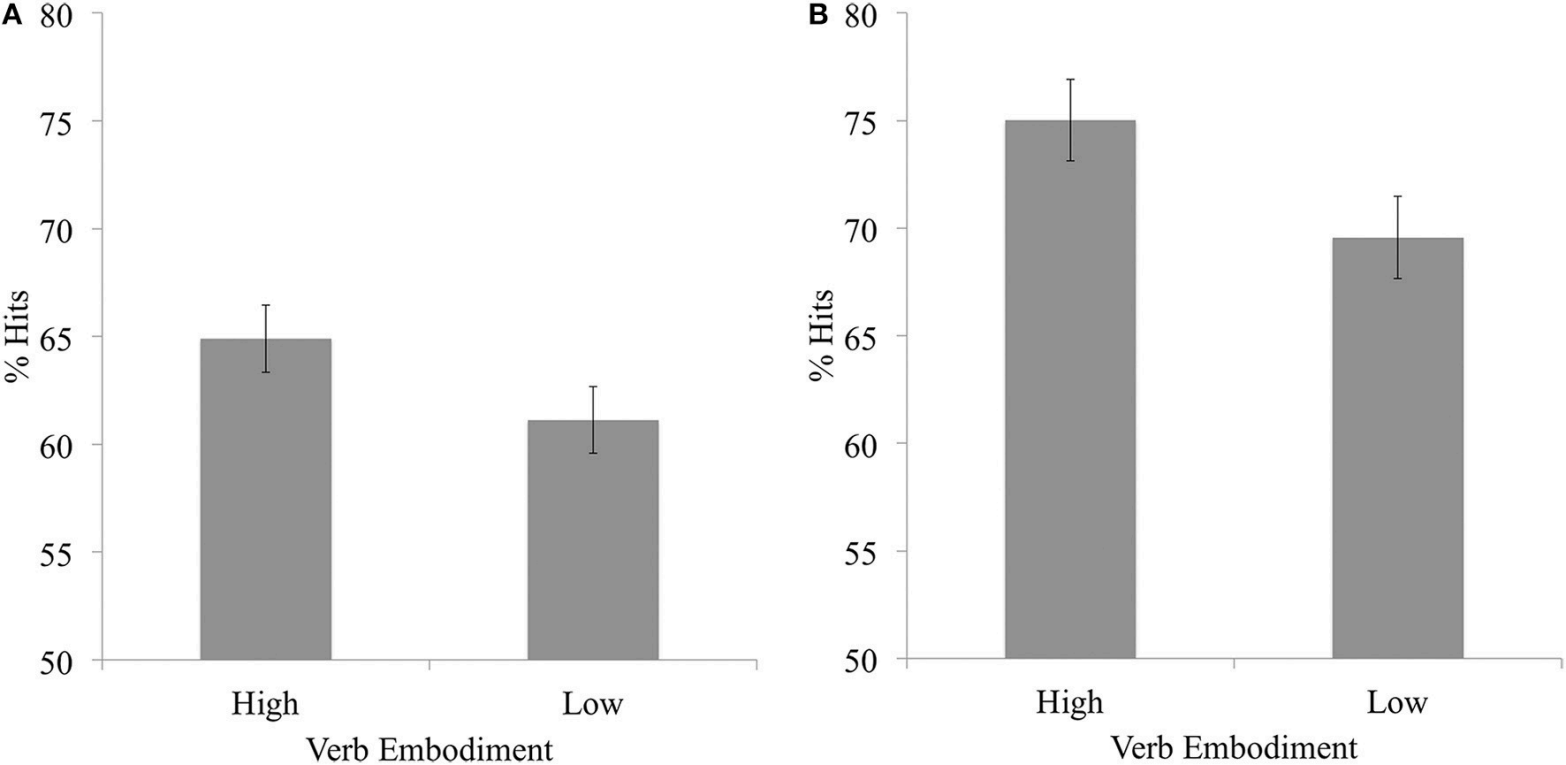
**Mean percentage of high and low embodiment items correctly identified as old (i.e., hits), in Experiment 1a (A) and 1b (B)**. Error bars represent 95% CIs using the Cousineau (2005) correction.

We used paired-samples *t*-tests to examine if recognition memory performance differed for high and low embodiment verbs. Results indicated that the hit rate was significantly higher for high embodiment verbs (*M* = 64.90, *SD* = 18.78) than for low embodiment verbs (*M* = 61.12, *SD* = 18.69), *t*_(55)_ = 2.43, *p* = 0.02, Cohen's *d* = 0.33 (see Figure 1A). There was no significant difference between these verbs in false alarm rates, *t*_(55)_ = 1.62, *p* = 0.11; or *d'* score, *t*_(55)_ = 0.76, *p* = 0.45. Finally, we investigated if participants set a different response criterion *C* for each type of verb, using the following equation: *C* = −0.5 × (*z*Hit Rate + *z*False Alarm Rate) (Macmillan and Creelman, 1991). This revealed that participants set a significantly lower response criterion *C* for high embodiment verbs (*M* = 0.13, *SD* = 0.44) than for low embodiment verbs (*M* = 0.22, *SD* = 0.40), *t*_(55)_ = 3.18, *p* = 0.002, Cohen's *d* = 0.43. See Table 2.

**Table 2:** **Mean scores (standard deviations in parentheses) on memory measures for high and low embodiment verbs, in Experiments 1a and 1b**.

**Memory measure**	**High embodiment verbs**	**Low embodiment verbs**
**EXPERIMENT 1A**		
% Hits	64.90 (18.78)	61.12 (18.69)
% FAs	27.04 (15.19)	24.90 (12.62)
*d*' Score	1.09 (0.55)	1.04 (0.60)
Criterion *C*	0.13 (0.44)	0.22 (0.40)
**EXPERIMENT 1B**		
% Hits	75.03 (10.24)	69.56 (12.59)
% FA	28.81 (14.31)	30.19 (14.02)
*d*' Score	1.32 (0.51)	1.12 (0.52)
Criterion *C*	−0.05 (0.31)	0.00 (0.32)

#### Discussion

Participants were more likely to identify a verb as having been previously studied if it was high in embodiment. This may suggest that high embodiment verbs, and the simulations they elicit, prompt more elaborative encoding. Participants also set a significantly lower response criterion for high embodiment verbs, which may suggest that participants are sensitive to the amount of simulation evoked by an item, and that this may affect how much evidence they require before identifying a target as being previously seen.

### Experiment 1b

#### Method

##### Participants

Participants were 32 undergraduate students (28 female; *M* age = 20.91, *SD* = 5.21) at the University of Calgary who participated for bonus credit in a Psychology course.

##### Materials and Procedure

The stimuli and procedure were identical to Experiment 1a except for the following changes. Stimuli presented in the encoding task now remained onscreen for 3000 ms. This change was made in response to participant feedback that the longer 5000 ms encoding time Experiment 1a led to difficulty concentrating toward the end of the experiment. In addition, participants were tested in our laboratory, and made their responses via button press on a response box. Button assignment was counterbalanced across participants. Finally, the list of verbs that was used as old or new items (i.e., which of the lists constituted the studied items and which constituted the lures) was counterbalanced across participants.

#### Results

Paired-samples *t*-tests indicated that hit rate was again significantly higher for high embodiment verbs (*M* = 75.03, *SD* = 10.24) than for low embodiment verbs (*M* = 69.56, *SD* = 12.59), *t*_(31)_ = 2.93, *p* = 0.006, Cohen's *d* = 0.53 (see Figure 1B). There was no significant difference between high and low embodiment verbs in false alarm rates, *t*_(31)_ = 0.91, *p* = 0.37. However, *d'* scores were significantly larger for high embodiment verbs (*M* = 1.33, *SD* = 0.51) than for low embodiment verbs (*M* = 1.12, *SD* = 0.52), *t*_(31)_ = 3.00, *p* = 0.005, Cohen's *d* = 0.53. Finally, there was no significant difference between these verbs in terms of response criterion *C, t*_(31)_ = 1.26, *p* = 0.22. See Table 2.

#### Discussion

The results of Experiment 1b provide evidence of more accurate recognition memory for high embodiment verbs, in the form of a higher hit rate and *d'* for these items. Again, this may indicate that high embodiment verbs enjoy more elaborative encoding than low embodiment verbs.

## Experiment 2

Having found a memory benefit for high embodiment verbs, we further explored this phenomenon by examining the potential for interaction with the imagined enactment effect. While the enactment effect is well-documented, previous studies have not taken into account the amount of motor information that to be remembered actions elicit. We investigated whether instructions to simulate the actions might erase the benefit for high embodiment verbs. That is, encouraging the simulation of actions might nullify the relative difference in automatic simulation elicited by high and low embodiment verbs. Another possibility is that the advantage provided by instructing participants to simulate actions might be especially beneficial for high embodiment verbs, because of the greater amount of associated motor information that one could evoke. These results (i.e., an interaction) would suggest that embodiment and imagined enactment effects have the same underlying source. Conversely, we may observe separate effects for each that do not interact. This would suggest that the two effects stem from separate sources. By examining the interplay between this deliberate (enactment) simulation and automatic simulation, we hoped to learn more about both processes. We examined the effects of imagined enactment on both recognition (Experiment 2a) and recall memory (Experiment 2b). Although several studies have demonstrated a larger effect of enactment on recognition memory (e.g., Mohr et al., 1989), others have found a larger effect of enactment on recall memory (e.g., Svensson and Nilsson, 1989; Nilsson and Craik, 1990).

### Experiment 2a

#### Method

##### Participants

Participants were 44 undergraduate students (20 female; *M* age = 20.61, *SD* = 2.66) at the University of Calgary who participated for bonus credit in a Psychology course.

##### Materials and Procedure

Stimuli were 120 verbs selected from the embodiment norms collected by Sidhu et al. (2014). These verbs were separated into four lists of 30 items, each containing 15 verbs rated high in relative embodiment (i.e., had a rating > 3.5), and 15 verbs rated low in relative embodiment (i.e., had a rating < 3.5). Within each list, verbs high and low in embodiment were again matched in terms of length, log transformed HAL word frequency, number of morphemes (Balota et al., 2007), orthographic Levenshtein distance (Yarkoni et al., 2008), imageability (Chiarello et al., 1999), and age of acquisition (Kuperman et al., 2012); they did, however, differ in terms of their rated embodiment (Sidhu et al., 2014), see Table 3 Across lists, high and low embodiment verbs were also matched on all of these variables. Of the four lists, two pairs were yoked such that they appeared together as either studied items or lures. See Appendix B for a full list of verb stimuli.

**Table 3:** **Mean values (standard deviations in parentheses) of lexical and semantic variables for high and low embodiment verbs, Experiments 2a–3b**.

	**High embodiment verbs**	**Low embodiment verbs**	***t***	***p***	**Cohen's *d***
Length	5.73 (1.04)	5.88 (0.99)	0.81	0.42	0.15
Log HAL frequency	7.79 (1.49)	8.10 (1.45)	1.19	0.24	0.21
Number of morphemes	1.45 (0.50)	1.43 (0.50)	0.18	0.86	0.04
OLD	1.98 (0.34)	2.02 (0.34)	0.65	0.52	0.12
Imageability	407.35 (59.30)	391.73 (62.81)	1.40	0.16	0.26
AoA	8.77 (1.97)	9.18 (1.92)	1.15	0.25	0.21
Embodiment	4.34 (0.44)	2.78 (0.26)	23.57	<0.001	4.46

*OLD, Orthographic Levenshtein distance; AoA, Age of Acquisition*.

During the study phase, participants were shown verbs one at a time, in two blocks that differed in their instructions. These instructions were presented as strategies to help participants remember the verbs for an upcoming memory test. In one block participants were asked to imagine themselves performing the action described, in particular how it would *feel*. In this block verbs were shown in a second person, present tense phrase (e.g., *You are running*). In the other block, participants were asked to imagine seeing someone else perform the action described as though watching a film. In this block verbs were shown in a first person, present tense phrase (e.g., *I am running*). These pronouns were based on those used by Ditman et al. (2010) to elicit either simulations of the reader performing an action (i.e., second person phrases) or watching another performing an action (i.e., first person phrases). In contrast to typical studies on the enactment effect, we only presented participants with verbs as opposed to verb-noun phrases because our interest was specifically in the effects of rated embodiment of each verb. Each block contained one of the four lists of verbs (i.e., each block contained 15 high embodiment verbs and 15 low embodiment verbs). Block order was counterbalanced across participants, as was the assignment of list to block type. Each trial began with a fixation cross that remained onscreen for 500 ms. This was replaced by the phrase, which remained onscreen for 3000 ms. Finally, the phrase was replaced by a 1000 ms blank screen before advancing to the next trial.

Following completion of the study phase, participants were asked to solve as many multiplication problems as they could in 5 min, after which they took part in a recognition test. During the test phase, participants saw both of the 30-item lists of verbs that they had studied earlier, as well as the two lists of 30 verbs that they had not studied, intermixed in a random order. Verbs were presented one at a time, on their own, in their infinitive form (e.g., *running*). Participants indicated whether the verb was old or new via button press on a response box. Button assignment was counterbalanced across participants, as was the assignment of lists as studied items or lures.

#### Results

Except for the analysis of false alarms, we used within subjects two-factor ANOVAs to examine if memory performance was affected by verb embodiment (high vs. low) and imagined enactment condition (imagine moving vs. imagine watching). Because enactment was not manipulated for false alarms (i.e., at test lures could not vary in enactment), those data were analyzed using a paired-samples *t*-test. In addition, when calculating *d'* score and criterion *C*, hit rates were compared to the false alarm rate for either high or low embodiment verbs, but could not be compared to false alarms at the same level of enactment.

**Figure 2:**
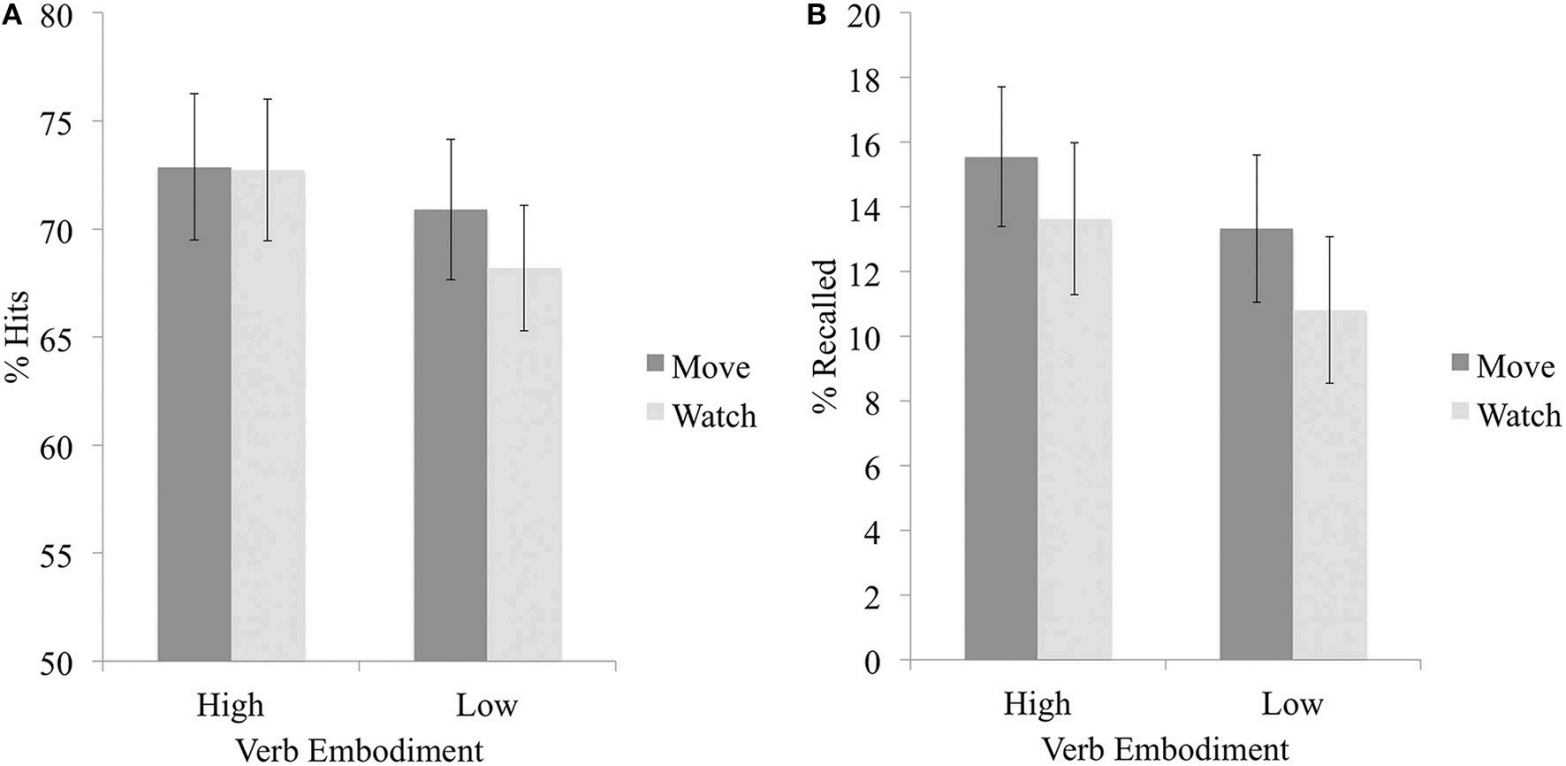
**Mean percentage of high and low embodiment items, encoded in the imagine moving or imagine watching blocks, that were correctly identified as old (i.e., hits), in Experiment 2a (A), or correctly recalled in Experiment 2b (B)**. Error bars represent 95% CIs using the Cousineau (2005) correction.

Results indicated a main effect of embodiment on hit rate, *F*_(1, 43)_ = 6.16, *p* = 0.02, η^2^ = 0.02; hit rate was again significantly higher for high embodiment verbs (*M* = 72.80, *SD* = 19.90) than for low embodiment verbs (*M* = 69.54, *SD* = 19.17). There was no main effect of imagined enactment on hit rate, *F*_(1, 43)_ = 0.32, *p* = 0.57; nor was there an interaction between embodiment and imagined enactment, *F*_(1, 43)_ = 0.66, *p* = 0.42 (see Figure 2A). There was no difference between high and low embodiment verbs in false alarm rates, *t*_(43)_ = 1.24, *p* = 0.22. Analysis of *d'* scores showed no main effects of either embodiment, *F*_(1, 43)_ = 0.43; or imagined enactment, *F*_(1, 43)_ = 0.40, *p* = 0.53; nor an interaction, *F*_(1, 43)_ = 2.30, *p* = 0.14. Lastly, results indicated a main effect of embodiment on criterion *C, F*_(1, 43)_ = 5.20, *p* = 0.03, η^2^ = 0.04; criterion *C* was significantly lower for high embodiment verbs (*M* = −0.14, *SD* = 0.52) than for low embodiment verbs (*M* = 0.07, *SD* = 0.38). There was no main effect of imagined enactment on criterion *C, F*_(1, 43)_ = 0.40, *p* = 0.53; nor was there an interaction between embodiment and imagined enactment, *F*_(1, 43)_ = 2.30, *p* = 0.14. See Table 4.

**Table 4:** **Mean scores (standard deviations in parentheses) on memory measures for high and low embodiment verbs, encoded in the imagine moving or imagine watching blocks, in Experiments 2a and 2b**.

**Memory measure**	**Imagine moving**		**Imagine watching**	
	**High embodiment verbs**	**Low embodiment verbs**	**High embodiment verbs**	**Low embodiment verbs**
**EXPERIMENT 2A**				
% Hits	72.87 (22.83)	70.90 (21.51)	72.72 (16.99)	68.18 (16.82)
% FAs	34.32 (23.51)^a^	32.81 (24.18)^a^		
*d*' Score	1.27 (1.58)	1.32 (1.53)	1.31 (1.27)	1.12 (1.13)
Criterion *C*	−0.13 (0.50)	−0.07 (0.50)	−0.15 (0.54)	0.03 (0.47)
**EXPERIMENT 2B**				
% Recalled	15.56 (9.43)	13.33 (10.92)	13.63 (10.73)	10.81 (9.02)

a *False alarm rates refer to new high and low embodiment items as a whole, as new items could not vary in terms of imagined enactment*.

#### Discussion

As in the previous experiments, we observed a higher hit rate for high embodiment verbs as compared to low embodiment verbs. Notably, this persisted here despite instructions for participants to imagine themself or another person performing each action. We also observed that participants set a lower response criterion for these high embodiment items. However, there was no main effect of imagined enactment on any of these memory measures, nor was there any evidence that imagined enactment interacted with embodiment.

### Experiment 2b

#### Method

##### Participants

Participants were 45 undergraduate students (35 female; *M* age = 23.42, *SD* = 9.40) at the University of Calgary who participated for bonus credit in a Psychology course.

##### Materials and Procedure

The stimuli and procedure were identical to Experiment 2a, except that participants now took part in a recall test as opposed to a recognition test. On the recall test, they were asked to recall as many verbs as they could in 3 min.

#### Results

Intrusions were defined as recalled items not present on a participant's study list, or studied items that had been previously recalled by a participant (i.e., the same item recalled more than once). These made up an average of 28.64% (*SD* = 24.15) of items recalled by participants and were excluded from analyses. We used within-subjects two-factor ANOVAs to examine if correct memory performance was affected by verb embodiment (high vs. low) and imagined enactment condition (imagine moving vs. imagine watching). Results indicated a main effect of embodiment on recall accuracy, *F*_(1, 44)_ = 5.04, *p* = 0.03 η^2^ = 0.03; a larger percentage of the high embodiment verbs were correctly recalled (*M* = 14.59, *SD* = 7.73) as compared to the low embodiment verbs (*M* = 12.07, *SD* = 7.56). There was no main effect of imagined enactment, *F*_(1, 44)_ = 2.19, *p* = 0.15; nor was there an interaction between embodiment and imagined enactment, *F*_(1, 44)_ = 0.06, *p* = 0.81 (see Figure 2B). See Table 4.

#### Discussion

We observed higher recall accuracy for high embodiment verbs, suggesting that the memory benefit observed for these items on recognition memory also extends to recall memory. However, once again, we did not observe an effect of imagined enactment, nor an interaction between imagined enactment and embodiment.

## Experiment 3

In order to try and make the imagined enactment manipulation more salient, we next modified the phrases in the imagine watching condition, using a third person as opposed to first person pronoun (Experiment 3a). Although Ditman et al. (2010) observed a larger effect for first person than third person phrases (relative to second person phrases), recall that those phrases were embedded in longer scenarios. It seemed possible that when presented in isolation, the first person phrases may have been confusing for participants, and perhaps led them to imagine *themselves* performing the actions in both conditions.

We further modified the imagined enactment conditions in Experiment 3b in order to address the concern that participants may have inadvertently imagined themselves performing the actions in the imagine watching condition. It is well-documented that individuals will simulate themselves performing an action that they perceive another person performing (e.g., Buccino et al., 2001). To circumvent this, and to again try to make the imagined enactment manipulation more salient, we instructed participants to imagine an inhuman robot performing the actions in the imagine watching condition.

### Experiment 3a

#### Method

##### Participants

Participants were 48 undergraduate students (45 female; *M* age = 20.35, *SD* = 4.53) at the University of Calgary who participated for bonus credit in a Psychology course.

##### Materials and Procedure

The stimuli and procedure were identical to Experiment 2b, except for the block in which participants imagined watching another person performing the action. Verbs in this block were now shown in a third person, present tense phrase (e.g., *He/She is running*). Pronoun gender was matched to reported participant gender.

#### Results

**Figure 3:**
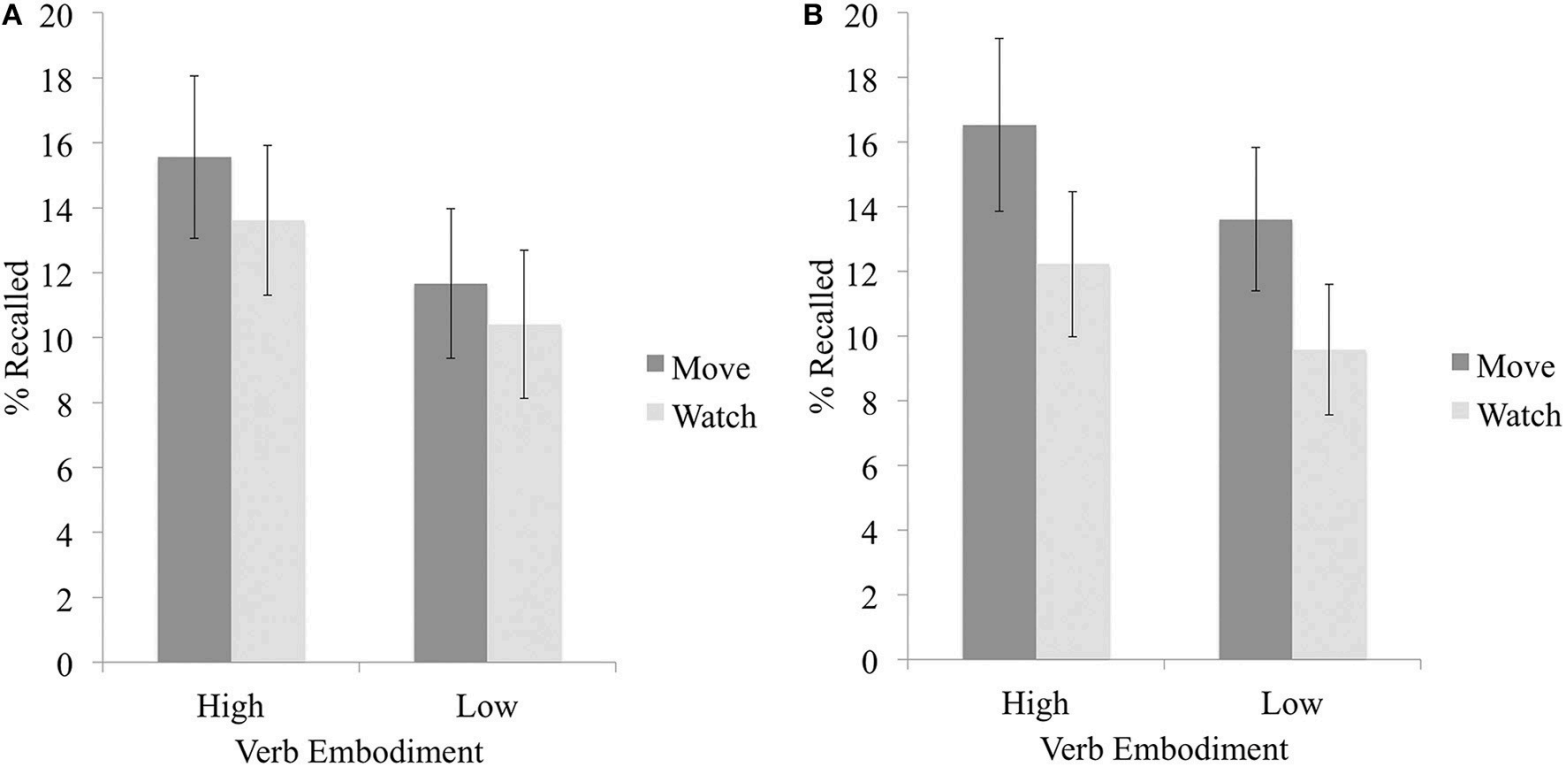
**Mean percentage of high and low embodiment items, encoded in the imagine moving or imagine watching blocks, that were correctly recalled in Experiment 3a (A) or 3b (B)**. Error bars represent 95% CIs using the Cousineau (2005) correction.

Intrusions made up an average of 24.18% (*SD* = 21.70) of items recalled by participants and were excluded from analyses. Results again indicated a main effect of embodiment on recall accuracy, *F*_(1, 47)_ = 7.89, *p* = 0.007, η^2^ = 0.05; a larger percentage of the high embodiment verbs were correctly recalled (*M* = 14.58, *SD* = 9.01) as compared to the low embodiment verbs (*M* = 11.04, *SD* = 7.18). There was no main effect of imagined enactment, *F*_(1, 47)_ = 1.59, *p* = 0.21; nor was there an interaction of embodiment and imagined enactment, *F*_(1, 47)_ = 0.05, *p* = 0.82 (see Figure 3A).

#### Discussion

Once again, we observed a benefit for high embodiment verbs on recall memory, but no effect of imagined enactment, nor an interaction with embodiment.

### Experiment 3b

#### Method

##### Participants

Participants were 48 undergraduate students (42 female; *M* age = 20.63, *SD* = 3.85) at the University of Calgary who participated for bonus credit in a Psychology course.

##### Materials and Procedure

The stimuli and procedure were identical to those in Experiment 3a, except for the block in which participants imagined watching another person performing the action. Participants were now asked to imagine watching a non-human robot performing the action, in particular R2-D2^1^ from the Star Wars movie franchise (see Appendix C for verbatim instructions). The instructions for this block were presented with an image of R2-D2; verbs in this block were shown in a third person, present tense phrase (e.g., *R2-D2 is running*).

#### Results

Intrusions made up an average of 31.13% (*SD* = 25.04) of items recalled by participants and were excluded from analyses. Results again indicated a main effect of embodiment on recall accuracy, *F*_(1, 47)_ = 7.51, *p* = 0.009, η^2^ = 0.03; a larger percentage of the high embodiment verbs were correctly recalled (*M* = 14.38, *SD* = 7.47) as compared to the low embodiment verbs (*M* = 11.60, *SD* = 7.99). Importantly, there was also a main effect of imagined enactment, *F*_(1, 47)_ = 6.59, *p* = 0.014, η^2^ = 0.06; a larger percentage of the verbs encoded during the imagine moving block were correctly recalled (*M* = 15.07, *SD* = 9.43) as compared to those encoded during the imagine watching block (*M* = 10.90, *SD* = 8.33). There was again no interaction between embodiment and imagined enactment, *F*_(1, 47)_ = 0.01, *p* = 0.91 (see Figure 3B; Table 5). We investigated the degree of evidence supporting this lack of an interaction using a Bayesian approach, and calculated the relative posterior probabilities of the null and alternate hypotheses (Masson, 2011). This revealed positive evidence (Raftery, 1995) that the interaction was not likely to exist, *p*_BIC_(H_0|_D) = 0.92; *p*_*BIC*_(H_1|_D) = 0.08.

**Table 5:** **Mean scores (standard deviations in parentheses) on memory measures for high and low embodiment verbs, encoded in the imagine moving or imagine watching blocks, in Experiments 3a and 3b**.

**Memory measure**	**Imagine moving**		**Imagine watching**	
	**High embodiment verbs**	**Low embodiment verbs**	**High embodiment verbs**	**Low embodiment verbs**
**EXPERIMENT 3A**				
% Recalled	15.56 (11.53)	11.67 (9.58)	13.61 (11.34)	10.42 (9.91)
**EXPERIMENT 3B**				
% Recalled	16.53 (11.59)	13.61 (11.08)	12.22 (9.85)	9.58 (9.32)

#### Discussion

Consistent with the previous experiments, we observed a memory benefit for high embodiment verbs. For the first time, we also observed an imagined enactment effect such that participants had higher recall accuracy for verbs encoded in the imagine moving condition as opposed to the imagine watching (R2D2) condition. There was no interaction between factors, suggesting that these effects may rely on distinct processes.

## General discussion

A central proposal of embodied cognition is that sensorimotor information is involved in simulation, and that it plays an important role in language processing (Barsalou, 1999, 2008). As such, there may be language processing differences for words that elicit varying amounts of simulation. These differences would be consistent with the semantic richness literature, in which words with a greater amount of associated information enjoy processing benefits on a variety of lexical tasks (e.g., Pexman et al., 2008). Sidhu et al. (2014) examined body-based semantic richness in the context of verbs that varied in the extent to which they involved the human body (i.e., their relative embodiment) and found that high embodiment verbs were processed faster in several tasks. This was ostensibly due to the greater amount of sensorimotor simulation and thus semantic activation for these words. Here we sought to examine the effects of variation in simulation on memory.

In Experiments 1a-2a participants studied high and low embodiment verbs and then had their recognition memory tested. Hit rate was consistently higher for high as compared to low embodiment verbs. Experiments 2b-3b employed a recall test and results showed that participants also had higher recall accuracy for high embodiment verbs. These results are consistent with the proposal that items with a greater amount of associated bodily information enjoy more elaborate encoding, potentially due to a greater amount of evoked simulation. This more elaborate encoding then leads to a stronger memory trace for high embodiment items and facilitates memory performance. Thus, these results fit with previous results showing that item level variation can lead to differences in elaboration and memory (e.g., Hargreaves et al., 2012). It is possible that simulation may play a role in some of these effects, such as the memory benefit for animate over inanimate nouns (Bonin et al., 2014; the authors themselves mention this possibility), or for concrete over abstract words (e.g., Hamilton and Rajaram, 2001).

Interestingly, we found no effect of embodiment on false recognition of new items (i.e., no difference in false alarm rate). This result differs from that of Montefinese et al. (2013) who found a *higher* false alarm rate for manipulatory verbs. They speculated that this was due to these items sharing a category with previously seen manipulatory verbs, based on their shared motor information (i.e., all movements performed with the hands). However, in our experiments the high embodiment verbs did not share specific motor information, but rather shared a greater amount of embodiment in general. This would suggest that the higher false alarm rate observed by Montefinese et al. was indeed due to the overlapping motor information among manipulatory items, and that this is not a necessary outcome for high embodiment items. This is somewhat surprising given studies on motor fluency and false recognition (e.g., Yang et al., 2009). Yang et al. found that participants were more likely to falsely recognize letter dyads if they could be typed with a single hand. They interpreted this as being due to more fluent simulation for these items. With this in mind, we might have expected high embodiment items to be falsely recognized as old more often, due to the fact that it is easier to simulate the actions to which they refer. The fact that we did not observe an effect in false recognition suggests that different processes are likely involved for letter dyad recognition (the Yang et al. study) and word recognition (the present study).

Another interesting result was that in the present Experiments 1a and 2a (but not Experiment 1b), participants set a significantly lower response criterion for high embodiment verbs. One interpretation is that participants required less evidence of having previously seen high embodiment verbs before categorizing them as old; low embodiment verbs required more evidence. Previous research suggests that participants may be able to shift their response criterion on a trial-by-trial basis, however, this process seems to occur over several trials (e.g., Brown and Steyvers, 2005). The results presented here could suggest that participants set a lower response criterion on trials that elicit a greater amount of simulation. This possibility should be examined more systematically in future research.

The other aim of this paper was to examine the interplay between automatic simulation and the more deliberate simulation evoked in the imagined enactment effect. In particular, we examined whether their effects on memory have the same or different sources. An imagined enactment effect proved difficult to observe, only appearing in one of four experiments. A potentially important difference between the present study and previous studies on the enactment effect is that participants in the present study only studied phrases containing actions as opposed to actions and nouns. In fact, some theorists point to a facilitated integration between actions and nouns as being responsible for the enactment effect (Kormi-Nouri, 1995). Another important difference between the present study and the one conducted by Ditman et al. (2010) is that in the latter, action phrases were embedded in larger scenarios. Thus, our results may suggest that imagined enactment effects are less robust when target items are presented in isolation. As to why an imagined enactment effect *did* emerge in Experiment 3b, our interpretation has to do with the actor being visualized in that imagine watching condition (i.e., an inhuman robot vs. a person). Perhaps it is difficult to avoid evoking some motor information when imagining another *person* performing an action, but it is less difficult to avoid this when imagining an inhuman robot performing an action. This combined with the previously mentioned differences may have made the imagined enactment manipulation too subtle to generate an effect in Experiments 2a-3a. It may also be worth noting that in the study conducted by Ditman et al. (2010), participants were not explicitly told to imagine performing or watching the actions described.

The most important finding from Experiment 3b was that when we did observe an imagined enactment effect, it did not interact with verb embodiment. On the contrary, these two factors appeared to have separate effects. Thus, it would be consistent with additive-factors logic to conclude that these two factors affected separate stages in processing (Sternberg, 1969). The effects of embodiment on memory have been assumed to result from differences between items in the automatic simulation of motor information (e.g., Madan and Singhal, 2012). Conversely, purposefully imagining the performance of an action likely involves a more deliberate process of simulation. It seems likely that the effects of these two processes on memory are distinct.

It is worth revisiting the results of Madan and Singhal (2012) who did not find a benefit for highly manipulable items when participants made responses based on the manipulability of items; in fact, under those conditions participants showed a memory benefit for non-manipulable items. The authors theorized that the deliberate process of responding to the manipulability question may have overridden the automatic memory enhancing effect of highly manipulable items, and thus allowed for task demands to play a role (e.g., making non-manipulable items stand out by virtue of their incongruence with the decision criterion). While these results are somewhat different than those observed in the present Experiment 3b, they point to a similar conclusion: automatic and deliberate simulation can have different effects.

Broadly, the memory benefit we observed for high embodiment verbs is consistent with the notion that these items elicited a greater amount of automatic simulation. Greater simulation likely led to more elaborate encoding for high embodiment items. Thus, while other contributions to the second edition of the *Meaning in mind: Semantic richness effects in language processing* Research Topic have demonstrated an advantage for semantically rich words on lexical processing tasks (e.g., Johns et al., 2016; Sidhu et al., 2016), the present results show that item level variation in semantic richness can also result in an advantage for memory. That is, our findings suggest that differences in the amount of simulation evoked can have effects beyond lexical processing tasks (e.g., Siakaluk et al., 2008; Sidhu et al., 2014), with implications for memory as well. While item-specific semantic richness effects in memory tasks have been reported in previous studies for noun stimuli (e.g., Hargreaves et al., 2012, in the first edition of the *Meaning in mind* Research Topic), those effects are extended here to verb stimuli. As such, our findings point to the generality of semantic richness effects, across paradigms and word classes.

## Author contributions

DS designed and ran the experiments and analyzed the data. PP helped design the experiments and interpret the data. DS wrote the first draft of the manuscript and PP helped modify the manuscript.

### Conflict of interest statement

The authors declare that the research was conducted in the absence of any commercial or financial relationships that could be construed as a potential conflict of interest.

## Footnotes

^1^The authors are aware that R2-D2 is in fact a droid (Sansweet, 1998), but will refer to him as a *robot* for the benefit of readers not familiar with the term *droid*.

## Appendix A

**Table:**

**HIGH EMBODIMENT VERBS IN EXPERIMENTS 1A AND 1B**		
Abuse	Descend	Meditate
Adapt	Detain	Molest
Align	Emerge	Nurture
Announce	Endure	Obstruct
Annoy	Evade	Pave
Arrange	Evolve	Pillage
Ascend	Excite	Pour
Assist	Exist	Produce
Attach	Expel	Pry
Attend	Expire	Punish
Beckon	Extend	Pursue
Bring	Flatten	Put
Build	Gather	Raise
Buy	Hasten	React
Cheat	Hinder	Recite
Communicate	Hiss	Relieve
Congregate	Hustle	Resist
Cram	Impair	Revive
Defend	Join	Roam
Depart	Learn	Sever
Deprive	Linger	Shut
Stalk	Suffer	Vacate
Steal	Swear	
Stop	Teach	
**LOW EMBODIMENT VERBS IN EXPERIMENTS 1A AND 1B**		
Absorb	Conduct	Excel
Accept	Confirm	Fade
Add	Deceive	Flourish
Adopt	Despise	Ignite
Adore	Detest	Insist
Aspire	Devote	Invent
Banish	Differ	Keep
Begin	Dilute	Lend
Believe	Divert	Lie
Belong	Divide	Melt
Betray	Dwell	Obey
Bless	Earn	Oppose
Borrow	Embark	Owe
Brighten	Enable	Promote
Cancel	Engage	Reflect
Choose	Enrich	Refrain
Commend	Evaluate	Regain
Comply	Evaporate	Reject
Condemn	Exceed	Renew
Repay	Sift	Unify
Resolve	Simmer	Wallow
Restore	Solve	Zoom
Revise	Spend	
Save	Sweeten	

## Appendix B

**Table:**

**HIGH EMBODIMENT VERBS IN EXPERIMENTS 2A–3B**		
Adapting	Evading	Pillaging
Aligning	Evolving	Pouring
Announcing	Exciting	Producing
Annoying	Existing	Prying
Arranging	Expelling	Punishing
Ascending	Expiring	Pursuing
Assisting	Extending	Raising
Attaching	Flattening	Reacting
Attending	Gathering	Reciting
Beckoning	Hastening	Relieving
Building	Hindering	Resisting
Cheating	Hissing	Reviving
Cramming	Hustling	Roaming
Defending	Impairing	Severing
Departing	Joining	Stalking
Depriving	Lingering	Stealing
Descending	Meditating	Suffering
Detaining	Nurturing	Swearing
Emerging	Obstructing	Teaching
Enduring	Paving	Vacating
**LOW EMBODIMENT VERBS IN EXPERIMENTS 2A–3B**		
Absorbing	Deceiving	Lending
Accepting	Despising	Opposing
Adding	Detesting	Promoting
Adopting	Devoting	Reflecting
Adoring	Differing	Refraining
Aspiring	Diluting	Regaining
Banishing	Diverting	Rejecting
Beginning	Dividing	Renewing
Belonging	Dwelling	Repaying
Betraying	Earning	Resolving
Blessing	Embarking	Restoring
Borrowing	Enabling	Revising
Brightening	Engaging	Sifting
Cancelling	Enriching	Simmering
Choosing	Exceeding	Solving
Commending	Fading	Spending
Complying	Flourishing	Sweetening
Condemning	Igniting	Unifying
Conducting	Insisting	Wallowing
Confirming	Inventing	Zooming

## Appendix c

Instructions for the Imagine Watching Condition in Experiment 3b. You will now see phrases describing actions performed by R2-D2 (pictured above). For each phrase, it's important that you imagine seeing R2-D2 performing the activity described. Imagine watching the activity as if it were in a video. Let the experimenter know if you have any questions. If not, press any button to begin.

## References

[B1] Balota D. A. Ferraro F. R. Connor L. T. (1991). On the early influence of meaning in word recognition: a review of the literature, in The Psychology of Word Meanings, ed Schwanenfllugel P. J. (Hillsdale, NJ: Erlbaum), 187–222.

[B2] Balota D. A. Yap M. J. Hutchison K. A. Cortese M. J. Kessler B. Loftis B.. (2007). The English lexicon project. Behav. Res. Methods 39, 445–459. 10.3758/BF03193014 17958156

[B3] Barsalou L. W. (1999). Perceptions of perceptual symbols. Behav. Brain Sci. 22, 637–660. 10.1017/S0140525X99532147 11301525

[B4] Barsalou L. W. (2008). Grounded cognition. Annu. Rev. Psychol. 59, 617–645. 10.1146/annurev.psych.59.103006.093639 17705682

[B5] Bonin P. Gelin M. Bugaiska A. (2014). Animates are better remembered than inanimates: further evidence from word and picture stimuli. Mem. Cognit. 42, 370–382. 10.3758/s13421-013-0368-8 24078605

[B6] Brown S. Steyvers M. (2005). The dynamics of experimentally induced criterion shifts. J. Exp. Psychol. Learn. Mem. Cogn. 31, 587–599. 10.1037/0278-7393.31.4.587 16060767

[B7] Brunyé T. T. Ditman T. Mahoney C. R. Augustyn J. S. Taylor H. A. (2009). When you and I share perspectives pronouns modulate perspective taking during narrative comprehension. Psychol. Sci. 20, 27–32. 10.1111/j.1467-9280.2008.02249.x 19076318

[B8] Buccino G. Binkofski F. Fink G. R. Fadiga L. Fogassi L. Gallese V.. (2001). Action observation activates premotor and parietal areas in a somatotopic manner: an fMRI study. Eur. J. Neurosci. 13, 400–404. 10.1046/j.1460-9568.2001.01385.x 11168545

[B9] Chiarello C. Shears C. Lund K. (1999). Imageability and distributional typicality measures of nouns and verbs in contemporary English. Behav. Res. Methods Instr. Comput. 31, 603–637. 10.3758/BF03200739 10633978

[B10] Cohen R. L. (1981). On the generality of some memory laws. Scand. J. Psychol. 22, 267–281. 10.1111/j.1467-9450.1981.tb00402.x

[B11] Connell L. Lynott D. (2015). Embodied semantic effects in visual word recognition, in Foundations of Embodied Cognition, Vol. 2, Conceptual and Interactive Embodiment, eds Coello Y. Fischer M. (Hove, UK: Psychology Press), 71–89.

[B12] Cousineau D. (2005). Confidence intervals in within-subject designs: a simpler solution to Loftus and Masson's method. Tutor. Quant. Methods Psychol. 1, 42–45.

[B13] Denis M. Engelkamp J. Mohr G. (1991). Memory of imagined actions: imagining oneself or another person. Psychol. Res. 53, 246–250. 10.1007/BF0094139427342256

[B14] Ditman T. Brunyé T. T. Mahoney C. R. Taylor H. A. (2010). Simulating an enactment effect: pronouns guide action simulation during narrative comprehension. Cognition 115, 172–178. 10.1016/j.cognition.2009.10.014 19939357

[B15] Engelkamp J. (1998). Memory for Actions. East Sussex: Psychology Press.

[B16] Engelkamp J. Krumnacker H. (1980). Image-and motor-processes in the retention of verbal materials. Zeitschrift Exp. Angewandte Psychol. 27, 511–533.

[B17] Engelkamp J. Zimmer H. D. Denis M. (1989). Paired associate learning of action verbs with visual-or motor-imaginal encoding instructions. Psychol. Res. 50, 257–263. 10.1007/BF00309262

[B18] Gallese V. (2007). Before and below ‘theory of mind’: embodied simulation and the neural correlates of social cognition. Philos. Trans. R. Soc. Lond B Biol. Sci. 362, 659–669. 10.1098/rstb.2006.2002 17301027 PMC2346524

[B19] Glenberg A. M. (2015). Few believe the world is flat: how embodiment is changing the scientific understanding of cognition. Can. J. Exp. Psychol. 69, 165–171. 10.1037/cep0000056 26010024

[B20] Glenberg A. M. Kaschak M. P. (2002). Grounding language in action. Psychon. Bull. Rev. 9, 558–565. 10.3758/BF03196313 12412897

[B21] Hamilton M. Rajaram S. (2001). The concreteness effect in implicit and explicit memory tests. J. Mem. Lang. 44, 96–117. 10.1006/jmla.2000.2749

[B22] Hargreaves I. S. Pexman P. M. Johnson J. C. Zdrazilova L. (2012). Richer concepts are better remembered: number of features effects in free recall. Front. Hum. Neurosci. 6:73. 10.3389/fnhum.2012.00073 22514526 PMC3322485

[B23] Hauk O. Johnsrude I. Pulvermüller F. (2004). Somatotopic representation of action words in human motor and premotor cortex. Neuron 41, 301–307. 10.1016/S0896-6273(03)00838-9 14741110

[B24] Helstrup T. (1986). Separate memory laws for recall of performed acts? Scand. J. Psychol. 27, 1–29.

[B25] Johns B. Sheppard C. L. Jones M. Taler V. (2016). The role of semantic diversity in word recognition across aging and bilingualism. Front. Psychol. 7:703 10.3389/fpsyg.2016.00703 PMC4937810 27458392

[B26] Kormi-Nouri R. (1995). The nature of memory for action events: an episodic integration view. Eur. J. Cogn. Psychol. 7, 337–363. 10.1080/09541449508403103

[B27] Kuperman V. Stadthagen-Gonzalez H. Brysbaert M. (2012). Age-of-acquisition ratings for 30,000 English words. Behav. Res. Methods 44, 978–990. 10.3758/s13428-012-0210-4 22581493

[B28] Lockhart R. S. Craik F. I. (1990). Levels of processing: a retrospective commentary on a framework for memory research. Can. J. Psychol. 44, 87–112. 10.1037/h0084237

[B29] Macmillan N. A. Creelman C. D. (1991). Signal Detection Theory: A User's Guide. Cambridge: Cambridge University Press.

[B30] Madan C. R. Singhal A. (2012). Encoding the world around us: motor-related processing influences verbal memory. Conscious. Cogn. 21, 1563–1570. 10.1016/j.concog.2012.07.006 22863476

[B31] Masson M. E. (2011). A tutorial on a practical Bayesian alternative to null-hypothesis significance testing. Behav. Res. Methods 43, 679–690. 10.3758/s13428-010-0049-5 21302025

[B32] Mohr G. Engelkamp J. Zimmer H. D. (1989). Recall and recognition of self-performed acts. Psychol. Res. 51, 181–187. 10.1007/BF00309146

[B33] Montefinese M. Ambrosini E. Fairfield B. Mammarella N. (2013). The “subjective” pupil old/new effect: is the truth plain to see? Int. J. Psychophysiol. 89, 48–56. 10.1016/j.ijpsycho.2013.05.001 23665094

[B34] Nilsson L. G. (2000). Remembering words and actions, in The Oxford Handbook of Memory, eds Tulving E. Craik F. I. M. (New York, NY: Oxford University Press), 137–148.

[B35] Nilsson L. G. Craik F. I. M. (1990). Additive and interactive effects in memory for subject-performed tasks. Eur. J. Cogn. Psychol. 2, 161–171. 10.1080/09541449008406210

[B36] Papesh M. H. (2015). Just out of reach: on the reliability of the action-sentence compatibility effect. J. Exp. Psychol. Gen. 144, e116–e141. 10.1037/xge0000125 26595844 PMC4662055

[B37] Pexman P. M. (2012). Meaning based influences on visual word recognition, in Visual Word Recognition, Vol. 2, ed Adelman J. S. (New York, NY: Psychology Press), 24–43.

[B38] Pexman P. M. Hargreaves I. S. Siakaluk P. D. Bodner G. E. Pope J. (2008). There are many ways to be rich: effects of three measures of semantic richness on visual word recognition. Psychon. Bull. Rev. 15, 161–167. 10.3758/PBR.15.1.161 18605497

[B39] Raftery A. E. (1995). Bayesian model selection in social research. Sociol. Methodol. 25, 111–163. 10.2307/271063

[B40] Sansweet S. J. (1998). The Star Wars Encyclopedia. New York, NY: Ballantine Books.

[B41] Siakaluk P. D. Pexman P. M. Aguilera L. Owen W. J. Sears C. R. (2008). Evidence for the activation of sensorimotor information during visual word recognition: the body–object interaction effect. Cognition 106, 433–443. 10.1016/j.cognition.2006.12.011 17258186

[B42] Sidhu D. M. Heard A. Pexman P. M. (2016). Is more always better for verbs? Semantic richness effects and verb meaning. Front. Psychol. 7:798. 10.3389/fpsyg.2016.00798 27303353 PMC4885847

[B43] Sidhu D. M. Kwan R. Pexman P. M. Siakaluk P. D. (2014). Effects of relative embodiment in lexical and semantic processing of verbs. Acta Psychol. 149, 32–39. 10.1016/j.actpsy.2014.02.009 24657828

[B44] Stanfield R. A. Zwaan R. A. (2001). The effect of implied orientation derived from verbal context on picture recognition. Psychol. Sci. 12, 153–156. 10.1111/1467-9280.00326 11340925

[B45] Sternberg S. (1969). The discovery of processing stages: extensions of Donders' method. Acta Psychol. 30, 276–315. 10.1016/0001-6918(69)90055-9

[B46] Svensson T. Nilsson L. G. (1989). The relation between recognition and cured recall in memory of enacted and nonenacted information. Psychol. Res. 51, 194–200. 10.1007/BF00309148

[B47] Tucker M. Ellis R. (1998). On the relations between seen objects and components of potential actions. J. Exp. Psychol. Hum. Percept. Perform. 24, 830. 9627419 10.1037//0096-1523.24.3.830

[B48] Wilson D. W. Golonka S. (2013). Embodied cognition is not what you think it is. Front. Psychol. 4:58. 10.3389/fpsyg.2013.00058 23408669 PMC3569617

[B49] Yang S. J. Gallo D. A. Beilock S. L. (2009). Embodied memory judgments: a case of motor fluency. J. Exp. Psychol. Learn. Mem. Cogn. 35, 1359–1365. 10.1037/a0016547 19686029

[B50] Yap M. J. Tan S. E. Pexman P. M. Hargreaves I. S. (2011). Is more always better? Effects of semantic richness on lexical decision, speeded pronunciation, and semantic classification. Psychon. Bull. Rev. 18, 742–750. 10.3758/s13423-011-0092-y 21494916

[B51] Yarkoni T. Balota D. Yap M. (2008). Moving beyond Coltheart's N: a new measure of orthographic similarity. Psychon. Bull. Rev. 15, 971–979. 10.3758/PBR.15.5.971 18926991

